# Dynamic Robustness of Open-Source Project Knowledge Collaborative Network Based on Opinion Leader Identification

**DOI:** 10.3390/e23091235

**Published:** 2021-09-21

**Authors:** Shaojuan Lei, Xiaodong Zhang, Suhui Liu

**Affiliations:** School of Economics and Management, University of Science and Technology Beijing, Beijing 100083, China; b20180404@xs.ustb.edu.cn (S.L.); b20170936@xs.ustb.edu.cn (S.L.)

**Keywords:** open-source project, identification of opinion leaders, SIR model, behavior degradation, robustness

## Abstract

A large amount of semantic content is generated during designer collaboration in open-source projects (OSPs). Based on the characteristics of knowledge collaboration behavior in OSPs, we constructed a directed, weighted, semantic-based knowledge collaborative network. Four social network analysis indexes were created to identify the key opinion leader nodes in the network using the entropy weight and TOPSIS method. Further, three degradation modes were designed for (1) the collaborative behavior of opinion leaders, (2) main knowledge dissemination behavior, and (3) main knowledge contribution behavior. Regarding the degradation model of the collaborative behavior of opinion leaders, we considered the propagation characteristics of opinion leaders to other nodes, and we created a susceptible–infected–removed (SIR) propagation model of the influence of opinion leaders’ behaviors. Finally, based on empirical data from the Local Motors open-source vehicle design community, a dynamic robustness analysis experiment was carried out. The results showed that the robustness of our constructed network varied for different degradation modes: the degradation of the opinion leaders’ collaborative behavior had the lowest robustness; this was followed by the main knowledge dissemination behavior and the main knowledge contribution behavior; the degradation of random behavior had the highest robustness. Our method revealed the influence of the degradation of collaborative behavior of different types of nodes on the robustness of the network. This could be used to formulate the management strategy of the open-source design community, thus promoting the stable development of OSPs.

## 1. Introduction

In contrast to a traditional product design process, the open-source design (OSD) community is spontaneously organized by diverse community members according to their interests and needs. To achieve common work goals for specific open projects, designers use collective wisdom, analyze knowledge, and carry out distributed task collaboration [1]. These designers often form a knowledge collaborative network (KCN) of a certain scale. This mass production mode is a bottom-up organization form, with low cost, strong flexibility, a high degree of innovation, and fast response; it has been successfully applied in software, encyclopedia, manufacturing, and commercial fields [2,3]. 

Similar to other forms of networks (e.g., social network, software network, business network, etc.), important nodes in the OSD community network—known as “opinion leaders”—dominate knowledge sharing, information dissemination, public opinion orientation, behavior, and decision-making guidance [4,5]. These opinion leaders transmit their views, ideas, models, and other information to other designers via their online community’s information communication channels, and this information is then radiated to the whole network. In this process, a single individual stimulated by endogenous reasons will imitate the behavior of some groups. The transmission phenomenon of this behavior is usually called the “following effect”. This complex propagation phenomenon exists in many areas, such as business and marketing [6], politics [7], public health [8], education [9], and social [10], among others. Owing to social influence and homogeneity [11], the opinion leaders’ characteristics of novelty, activity, and professional knowledge influence both the behavior of the other members of the community and decision making [12]. Opinion leaders play a key role in community management, public opinion guidance, and other decisions. Academics have studied the opinion leader identification model and the following effect in various online communities [13,14]. Most research perspectives only explore opinion leaders’ positive behaviors. However, when opinion leaders exhibit negative behaviors, such as a reduced willingness to collaborate, or even leaving the community, this also leads to the following effect of other nodes, and this has unexpected consequences on the network. There are many cases where the open-source community (OSC) has declined or failed due to the loss of a large number of members or the degradation of behavior [15,16]. Therefore, it is of great practical significance for the stable development of OSCs and open-source projects (OSPs) to conduct research on the robustness of KCNs in the face of the negative impacts caused by the negative behaviors of OSC opinion leaders.

## 2. Related Work

### 2.1. Opinion Leader Identification

Though the definition of opinion leaders differs between network types, all definitions reflect that opinion leaders have an influence on people’s attitudes, opinions, and behaviors [17,18]. In the OSC, the role of opinion leaders is not limited to information dissemination, public opinion guidance, and supervision; importantly, it also includes information processing, the effective dissemination of knowledge, and the promotion of collaboration [4]. Therefore, the identification of opinion leaders is one of the cores of community network research. The two common identification methods are the network structure analysis method and the information interaction analysis method [19]. The network structure analysis method (which is based on social network analysis) establishes an evaluation index system of opinion leaders that comprehensively considers the attributes, interactive behaviors, and topological information of users; it can comprehensively measure the importance and influence of users in the network. For example, Bonacich et al. [20] proposed to use degree value to show the importance of nodes. This index supposes that the greater the degree of nodes, the greater the importance of the nodes in the network. Freeman et al. [21] proposed a clustering analysis of users within the community based on betweenness, closeness, and flow betweenness as indicators. Ren et al. [22] proposed a network node importance measurement method based on the degree and clustering coefficient to effectively analyze the node importance of large-scale networks. Zhu Zhiguo et al. [23] combined the two aspects of “network centrality” and “user activity” to construct an index system of opinion leaders, and they ranked the opinion leaders with the grey correlation model. Ain et al. [24] used four different centrality metrics—closeness, betweenness, eigenvector, and PageRank—to sort the nodes in the network, then used Firefly algorithm to find local and global opinion leaders. Chen [25] built a social network using the attention relationship between individuals. They proposed a similarity evaluation method to identify opinion leaders by analyzing the behavior of users in the social network. The aforementioned evaluation indexes mainly consider the structure of the collaboration network but ignore the semantic content of the interaction and collaboration between users.

The information interaction analysis method identifies opinion leaders in the network using the semantic analysis of user comments or the sentiment analysis published by users. This method is more suitable and prevalent in social networks because it is mainly based on comment data. For example, Chen et al. [26] proposed a detection method of opinion leaders with positive and negative opinions, which built a signature network based on online comments. A new model based on negative trust was also designed to detect opinion leaders in communities. Jiang et al. [27] used MapReduce to design an opinion leader detection algorithm based on the improved PageRank algorithm. In this algorithm, the improved PageRank algorithm uses sentiment analysis to define the connection weight between users in a bulletin board system. 

Some scholars have created opinion leader identification models that combine the above two methods. For example, Clat [28] established a user influence model by combining content similarity and network topology. This method detects opinion leaders based on user influence and sentiment analysis. Li et al. [29] studied the microblogging community detection method based on semantic analysis and user relationship, applying a hierarchical clustering algorithm to search for opinion leaders. Wang [30] designed an opinion leader detection algorithm based on network structure and topic similarity; making use of user attributes, text characteristics, and topic similarity, this algorithm constructs a directed weighted network for social networks and uses the PageRank algorithm to mine opinion leaders. Ye and Du [31] proposed an opinion leader detection method based on network topology, user attributes, and sentiment analysis. The hierarchical structure is used for sentiment analysis to identify malicious users in the opinion leader set who pose a threat to national security. Atienza-Barthelemy et al. [32] established the directed weighted retweet network to study the relationship between ideology and language in the Catalan independence context. They carried out the selection of the users (opinion leaders) in two steps. The first step searched for the most influential, active, and engaged (continuous participation) users, and the second step was based on a hierarchical community analysis. Presently, research combining the two methods is also focused on social networks and content publishing communities such as Facebook, Twitter, and Weibo [33,34,35].

Since the combination of these two identification methods can identify opinion leaders from a more comprehensive index, we consider both the network structure and semantic content when identifying opinion leaders in the OSD community. However, compared with the content publishing community, the OSD community places more emphasis on knowledge collaboration. As such, there are many differences in the selection of identification indexes and the identification methods of opinion leaders. Therefore, this paper first proposes a semantic-based KCN construction method, to allow semantic information to be embodied in the KCN. Based on the constructed network, the opinion leader identification index considers the degree of interaction between nodes, that is, the ability of nodes themselves to influence other nodes. The communication location of nodes is considered from the perspective of the overall macrostructure of the network (i.e., the advantages of nodes in the KCN). For these indexes, an entropy weight and TOPSIS algorithm are used to evaluate the users in the community one by one to identify the opinion leaders.

### 2.2. The Propagation of Opinion Leader Behavior

As information and viruses spread in the network, the propagation of users’ behavior in the network follows certain rules. The traditional propagation model has great reference significance for the study of behavioral propagation in the network [36]. For example, Xu Bingcun et al. [4] set the opinion leaders (who were identified by the multi-attribute comprehensive evaluation method and the grey correlation method) as the initial source of infection and applied the susceptible–infected–removed (SIR) model to simulate the following behavior of users in the OSC. Zhang Weidong et al. [37] built a complete information game model to control the spread of false information by guiding users’ following behavior. Xiong et al. [38] divided Weibo users into three types based on the SIR model to simulate users’ “following” behavior. The above studies adopted the classical propagation model to study the propagation process of behaviors in the network, but they did not consider the influence of the propagation process on the overall performance of the network. Therefore, this paper adopts the classical SIR epidemic model to conduct a simulation experiment of propagation behavior dynamics on the changes in the overall network collaboration state caused by the following behavior of other nodes after opinion leader nodes reduce their willingness to collaborate. This verifies whether the key nodes identified above have a stronger influence on resource control, information propagation, and network stability. It also analyzes the change of network performance during the process of behavior propagation.

### 2.3. Complex Network Robustness

Robustness refers to the ability of the system to maintain its functions or characteristics when it is subjected to external interference or destruction [39]. Research on the robustness of complex networks originated from Albert et al. [40] in 2000, who mainly focused on the influence of topological structure on the destruction resistance of complex networks. Subsequently, many scholars have studied the robustness of different complex network structures (e.g., regular networks, random networks, small-world networks, and scale-free networks). Research content includes network structure, model parameters, degradation mode, robust decision making, and other aspects [41,42]. With the wide application of OSD in various industries, researchers are devoting increasing attention to the robustness and stability of OSC networks. For example, in their empirical study, Fuge et al. [43] took the online collaboration community OpenIdeo through a method of node attack and found that communities with cored-edge structures have strong robustness. Donadelli et al. [44] studied the impact of knowledge loss on OSC projects, finding that the departure of major contributors reduces the dissemination of knowledge and brings about disastrous effects on community projects. Zhou et al. [45] studied the impact of the intentional loss of knowledge contribution nodes and dissemination nodes on the robustness of OSC networks at different stages of development. Gamalielsson et al. [46] found that successful retention and further recruitment of contributors can improve the robustness of the open-source software community. Frank et al. [47] measured the departure and entry of nodes according to the cost–benefit relationship between active nodes in the community (i.e., when the cost exceeds the benefit, the node leaves the network). They studied the dynamic robustness of online social networks with the life cycle of core-periphery structure as the index. Lei et al. [48] analyzed the degree of network performance loss of OSD communities under different degradation modes of knowledge collaboration behavior from both a static and dynamic perspective.

We found that most current studies on the robustness of OSCs adopt the nodal-based degradation approach. When the nodes are deleted, the edges directly connected to the nodes are also deleted, resulting in the ineffective use of edge strength information. In OSCs, designer nodes will not withdraw directly from the project or community after negative interference; they will first be reflected in the decrease of collaboration intensity with other designer nodes. Additionally, due to the existence of social influence and homogeneity, the degradation of opinion leaders’ behavior in OSCs leads to the degradation of other nodes or edges. The influence of this behavior propagation phenomenon on network performance—where the degradation of individual nodes or edges causes the degradation of further nodes or edges—is rarely paid attention to.

In view of these problems, this paper first constructs a semantic-based OSC KCN by combining two key factors among designers: comment content and comment frequency. Four network features are selected and the entropy weight and TOPSIS evaluation method is adopted to identify the opinion leader nodes in the network. Then, based on the identified opinion leaders, the SIR epidemic model is applied to design the propagation mode of the degradation of opinion leaders’ collaborative behavior. Finally, robustness analysis is carried out using empirical data from the Local Motors open car design community. This includes analysis on the degradation of (a) opinion leaders’ collaborative behavior, (b) main knowledge contribution behavior, and (c) main knowledge dissemination behavior. Based on research results, we present suggestions for robustness protection.

## 3. Construction of Directed, Weighted, Semantic-Based Knowledge Collaborative Network

### 3.1. Semantic-Based Weight Calculation

In the OSD community, the large-scale collaborative behavior of designers (e.g., sharing, suggesting, evaluating, improving, etc.) is the key process to complete product design. To construct a directed, weighted, semantic-based KCN: (1) the knowledge collaboration behaviors of designers in the large-scale collaboration process are screened out, including semantic content at the knowledge production level and social behavior at the knowledge circulation level; (2) the semantic content at the knowledge production level is represented by collaborative content intensity$\;g_{i}$ and the social behavior at the knowledge circulation level is represented by collaborative frequency intensity $k_{i,j}$; and (3) the designers are taken as nodes, the collaborative behaviors between nodes are taken as edges, and the network edge weight$\;W_{i,j}$ is obtained by weighting the collaborative content intensity$\;g_{i}$ and the collaborative frequency intensity$\;k_{i,j}\;$between designers. The calculation of the edge weight of the network is:

(1) $$\left\{\begin{array}{c} W_{i,j}=\alpha g_{i}+\beta k_{i,j}\;\;\;\;\;\;\;\;k_{i,j}>0\\ 0\;\;\;\;\;\;\;\;\;\;\;\;\;\;\;\;\;\;\;\;\;\;\;\;\;\;\;\;\;\;\;\;\;\;\;\;\;\;\;\;\;k_{i,j}=0 \end{array}\right.$$

where α and β are the influence coefficients of the content intensity and frequency intensity, respectively, satisfying α+β=1. The collaboration frequency strength $k_{i,j}$ is obtained by normalizing the one-way collaboration times $k_{i,j}^{\prime }$ from designers i to j. The collaborative content strength $g_{i}$ of designers i is calculated by (1) using the RAKE algorithm [49] to extract keywords from the overall review text of the project and (2) calculating and normalizing the matching degree between designers’ collaboration content and project keywords. The calculation process of the collaborative content intensity$\;g_{i}$ is as follows.

1. Calculate the keyword candidate set $T^{\prime }$: Take all collaborative content contained in the community project as the target text, then take punctuation marks and stop words as word segmentation intervals to obtain a candidate set of text keywords, $T^{\prime }=\left\{t_{1},\;t_{2},\;t_{3},\ldots ,\;t_{z}\;\right\}\;$.
2. Construct the co-occurrence matrix$\;D_{zz}$: the frequency of occurrences of the candidate $t_{m}\;$in the text is$\;a_{m,m}$ and the frequency of co-occurrences of $t_{m\;}$and$\;t_{n}$ in the same phrase is$\;a_{m,n}$. The co-occurrence matrix $D_{zz}$ is:
  (2) $$D_{zz}=\left[\begin{array}{ccc} a_{1,1} & \cdots & a_{1,z}\\ \vdots & \ddots & \vdots \\ a_{z,1} & \cdots & a_{z,z} \end{array}\right]$$
3. Calculate the candidate word weight $W_{t_{m}}$: according to the co-occurrence matrix, obtain the degree of candidate word$\;t_{m}$: $Deg_{m}$ = $\overset{z}{\underset{n=1}{\sum }}a_{m,n}$, the frequency of candidate word $t_{m}$: $Feg_{m}=$ $a_{m,m}$, then use their ratio to represent the weight $W_{t_{m}}$ of candidate word $t_{m}$:
  (3) $$W_{t_{m}}=\frac{Deg_{m}}{Feg_{m}}=\frac{\sum_{n=1}^{z}a_{m,n}}{Feg_{m}}$$
  Arrange in descending order according to the calculated weight$\;W_{t_{m}}$. Take the candidate words in the top 1/3 of the ranking as the keywords of the text and output the keyword set T=t1, t2, t3,…, tz3  and the weight value$\;W_{t_{m}}$ of each keyword.
4. Calculate the content intensity of each designer: follow step (1) to segment the overall comment content of each designer in the project to obtain a set of keyword candidates for each designer $T_{i}=\left\{t_{1},\;t_{2},\;t_{3},\ldots \right\}$, then calculate $g_{i}{}^{\prime }$ as the sum of the weights of the keywords contained in the designer comment text:
  (4) $$g_{i}{}^{\prime }=\underset{t_{m}\in T\cap Ti}{\sum }W_{t_{m}}$$
  where: T is the keyword set, $T_{i}$ is the candidate word set of designer i , and $t_{m}$ is the keyword contained in designer i.
5. Obtain $g_{i}\;$by normalizing $g_{i}{}^{\prime }$.

### 3.2. KCN Structure Characteristics

In this paper, we used Local Motors, an OSC for car design, as the research object. The Local Motors community has an avant-garde design concept of “production for customers”, where designers can freely choose design projects of interest, exchange creative models, and propose design solutions. In this mode, the design scheme can be tested, retested, and perfected in this collaboration cycle. After finalization, it can be co-manufactured with the customers who buy the products.

We chose project LF-01, which has the largest number of participants and can best reflect the characteristics of this community, for our research. This project was established in January 2014, and as of 16 November 2016, the project contained 673 designers and 7757 instances of communication. The network features of LF-01 are consistent with most projects; therefore, it can suitably represent the situation of most OSPs.

To better simulate two-way dissemination and two-way collaboration behaviors among community members, this paper uses a directed weighted network graph to model. Community designers are represented by node set V and the comment relationship between designers is represented by edge set E. The direction of the edge is consistent with the direction of the comment, that is, if designer i comments on j, then the edge direction between them is i to j. According to the method introduced in Section 3.1, the edge weight is represented by the edge weight set W. In the calculation of the edge weight of the constructed network, the collaborative content intensity α and collaborative frequency β were considered equally important; they were each given a value of 0.5. After filtering out information that is unrelated to knowledge collaboration in the original data, it remains a total of 463 nodes, 3129 edges. Therefore, the semantic-based KCN model is$\;G\;=\left(V,\;E,\;W\right)$, where the larger the vertex strength in the network, the larger its area. Table 1 shows the topological parameters of the constructed network.

Table 1 shows that there are 463 nodes participating in knowledge collaboration in the network. The average out-degree and average path length of the network are relatively small, which indicates that the overall network structure is relatively sparse, the information mobility is not strong, and the information dissemination is insufficient. 

The small-world characteristic shows that the network has some “shortcut” connections to connect different subgroups, that is, there are some key collaborations in the knowledge collaborative relationship of many participants, and they play a key role in reducing the network distance. Therefore, it is necessary to study the opinion leaders in the network. The scale-free characteristic indicates that during the evolution of the project network, new participants tend to connect to larger nodes in the original network. The disassortative characteristic reflects that nodes with lower degree values are more inclined to establish connections with nodes with higher degree values. Therefore, it is also necessary to study the influence of the following effect on network performance.

## 4. Opinion Leader Identification Based on Entropy Weight and TOPSIS

Identifying opinion leaders in OSP can be seen as finding the most important nodes in the KCN. Generally, the importance of nodes can be measured by the centrality index [52]. The importance of a node is related to the overall structure of the network. It is necessary to make a comprehensive evaluation by using multiple important indicators of the node from different angles. Consequently, we propose a multi-attribute decision-making method based on the entropy weight and TOPSIS [53]. The multiple indexes of a single node (e.g., degree centrality, closeness centrality, betweenness centrality, structural hole, etc.) are taken as the attributes of the decision evaluation scheme for a comprehensive calculation to determine its importance in KCN. Considering multiple importance indicators, the comprehensive evaluation of nodes can cover a variety of factors affecting the importance of nodes, so we can obtain more accurate node importance evaluation results than using a single index [54].

### 4.1. Analysis of Identification Index

During the communication and collaboration of design projects, designers in the OSD community can influence other designers’ views and behaviors, and can even stimulate creativity through comments, so as to finally complete the product design process. This can be regarded as the ability of a network node to influence other nodes. In social network analysis, centrality is the most important index with which to evaluate node attributes. It is used to measure a node’s importance and collaboration ability with other nodes. There are three centralities to note: (1) strength centrality, that is, the sum of the edge weights of the node pointing to other nodes, which reflects the node’s active knowledge collaboration ability; (2) closeness centrality, that is, the reciprocal of the shortest path average value between nodes, which shows the ability of one node to get rid of the control of others; and (3) betweenness centrality, that is, the number of times a node acts as an “Intermediary“, which measures the ability of a node to control the collaboration with others. 

Further, opinion leaders are often in the structural hole position in the overall macrostructure of the network. They have more information resources and cross various groupuscule through knowledge collaboration. In this position, opinion leaders occupy the best communication location, which can filter information, disseminate information, and control the direction of public opinion [24]. Therefore, this paper presents four evaluation indexes: strength centrality, closeness centrality, betweenness centrality, and structural hole. We take the Local Motors OSD community as an example to illustrate the selection process of the four indexes.

1. Strength centrality.

The simplest measure of the centrality of a vertex in a network is just the degree of the vertex. In directed weighted networks, vertex strength is divided into in-strength and out-strength. The out-strength of node i is the sum of the edge weights of node i pointing to other nodes; in-strength refers to the sum of edge weights of other nodes pointing to node i [52]. Table 2 lists the respective out- and in-strengths of the top ten designer nodes of Local Motors’ OSP. The top ten designers have a high repetition rate and a small numerical difference, indicating that designers who prefer to collaborate with others usually have larger communication networks. In addition, even in the top ten, the increase in in-strength from minimum to maximum reached 67.07%, and the out-strength was as high as 76.95%. To integrate the in- and out-strengths of nodes, this paper will take the value after the symmetry of the network as the input data of the opinion leader identification model. This shows that the number of designers who occupy the leading position is very small; most designers are concerned about and follow the few influential designers.

**Figure 1:**
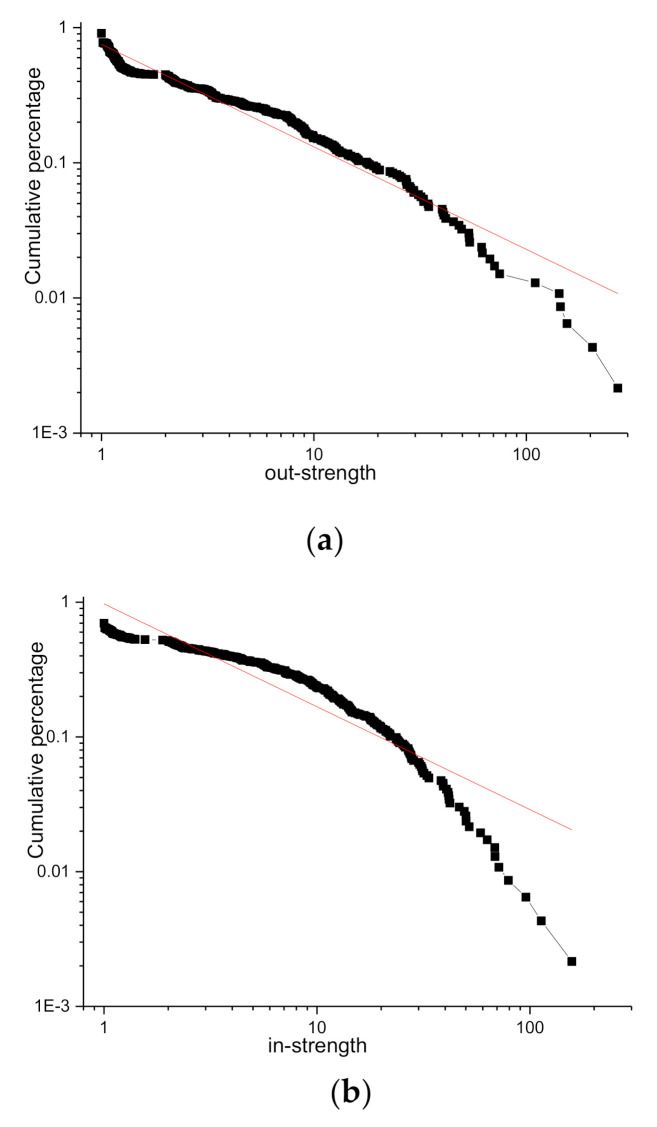
Cumulative distribution of out- and in-strength. Figure (**a**) shows the cumulative distribution of out-strength. Figure (**b**) shows the cumulative distribution of in-strength.

The cumulative distribution and fitting function of in- and out-strength are plotted in a logarithmic coordinate system, as shown in Figure 1. The cumulative distribution power exponent of in-strength is 2.62991 and the goodness of fit is$\;R^{2}=0.90327$; the cumulative distribution power exponent of out-strength is 4.86417 and goodness of fit is$\;R^{2}=0.94534$. The fitting effect of the power-law distribution of the strength of nodes is very good, which indicates that the connection between nodes in the network is irregular, and there is no accurate distance relationship, so it belongs to a scale-free network. Only a small number of designers participate in the main production element of the Local Motors OSC. It is, therefore, meaningful to identify the opinion leaders in the KCN.

2. Closeness centrality.

Closeness centrality is the reciprocal of the sum of the length of the shortest paths between the node and all other nodes in the network [55], and it measures the difficulty level of the node arriving at other nodes and the communication efficiency in the network. In-closeness is the shortest path from other nodes to this node, and out-closeness is the shortest path from this node to other nodes. Table 2 lists the respective closeness centralities of the top ten nodes of the Local Motors OSP. The in-closeness of the top ten nodes is around 0.4, which means they can easily communicate and collaborate with other designers in the network. The maximum value of 0.592 indicates that the network has a low concentration. Nodes with a closeness centrality near to 0 account for 47.2% of the total, indicating that nearly half of the designers are at an absolute disadvantage in the network’s information circulation. The general low out-closeness indicates that the efficiency of communication and collaboration of community designers is low, and the initiative needs to be improved. Therefore, we choose in-closeness as the input of the opinion leader identification model.

The top 10 nodes of in-closeness and out-closeness centrality are completely different, indicating that there are different types of designers in the network. For example, designers who have professional knowledge and are well known by others will choose more meaningful collaboration and have low initiative. Designers who actively seek collaboration cannot get an effective response. Therefore, it is necessary to identify the important nodes in the network.

3. Betweenness centrality.

Betweenness centrality is the number of short paths between nodes in the network that pass through a given node [56]. “Intermediary” means that nodes occupy a key position in the network dissemination path and play the role of disseminator. As shown in Table 2, of the top ten designer nodes in the Local Motors OSP, node 324 has the largest betweenness value, which is a very important bridge. The betweenness of other nodes in the top ten is also very high. Despite there being a large difference between them, they are still far higher than the 55.6% of nodes with 0 betweenness. Due to the following effect of most designers in the network, nodes with a large betweenness value are very important to maintaining the stability of the network.

Further observing Table 2, it can be found that the top five nodes with betweenness centrality are exactly the same as with the top five nodes with in-strength, and the coincidence degree with out-strength nodes has also reached 80%, indicating that users with strong collaboration ability also play an important “intermediary” role in the network.

4. Structural hole.

Structure hole mainly refers to the fact that there is no direct connection between the two nodes in the network, and they must be connected through a third party. The third party occupies the structural hole position in the relationship network and plays the role of “network bridge”. The existence of structural holes means the existence of opinion leaders.

In this paper, four structural hole indexes as proposed by Burt [57] are selected for analysis, which are effective size, efficiency, constraint, and hierarchy. Among them, the hierarchy and constraint are cost indexes, and effective size and efficiency are benefit indexes. (1) Effective size is the non-redundant information in the network. The larger the effective size, the lower the redundancy of network information and the greater the possibility of structural holes. (2) The node efficiency is equal to the ratio of the effective size of the node to the actual size. The higher the node efficiency, the higher the node activity efficiency in the network, and the faster the dissemination of information. (3) Constraint is the direct or indirect closeness between a node and other nodes in the network. The higher the constraint coefficient, the closer the node’s own-network (the node’s own-network refers to the network composed of the node and all nodes directly connected to it), and the less the number of structural holes. (4) Hierarchy indicates the degree to which all restrictions are concentrated on one node. The lower the hierarchy value, the more central the node’s position is and the greater the control power for the network is. For the Local Motors OSP, Table 3 lists the top ten nodes by the hierarchy value. We think that the hierarchy shows the degree of control of network information dissemination from the overall situation, and can better reflect its position in the overall network, so choose to input it into the opinion leader identification model.

### 4.2. Opinion Leader Identification Method

#### 4.2.1. Entropy Weight TOPSIS

In this paper, a node-importance synthesis method for multi-attribute decision making based on entropy weight and TOPSIS [54] is selected to identify opinion leaders. Each node is regarded as a decision scheme. The final evaluation is made by calculating the distance between each scheme and the optimal scheme. The specific steps for this are as follows.

1. The decision scheme set: $Q=\left\{Q_{1},Q_{2},\;\ldots \;Q_{m}\right\}$ consists of M nodes in the network. The set of N evaluation indexes: $U=\left\{U_{1},U_{2},\;\ldots \;U_{n}\right\}$;The jth index evaluation result of scheme i is$\;Q_{i}\left(U_{j}\right)\left(i=1,\;2,\;\cdots ,m;j=1,\;2,\;\cdots ,n\right)$.
2. Of the evaluation indexes selected in this paper, only the hierarchy in the structural hole is used as the cost index, and the others are used as the gain index. To eliminate the impact of different index dimensions, the evaluation results are normalized to$\;x_{ij}$, and then the original decision matrix is$\;X={\left(x_{ij}\right)}_{m\times n}$.
  (5) $$x_{ij}=\frac{Q_{i}\left(U_{j}\;\right)-Q_{i}{\left(U_{j}\;\right)}_{min}}{Q_{i}{\left(U_{j}\;\right)}_{max}-Q_{i}{\left(U_{j}\;\right)}_{min}}$$
  where: i=1, 2, ⋯,m;j=1, 2, ⋯,n, $Q_{i}{\left(U_{j}\;\right)}_{max}\;and\;Q_{i}{\left(U_{j}\;\right)}_{min}$ are the maximum and minimum values of the evaluation results respectively.
3. The entropy weight method is selected to determine the index weight. The weight of the jth evaluation index is expressed as $w_{j}$(j=1, 2, ⋯,n,$\;\sum w_{j}=1$). The weighted standard decision matrix Y is obtained as follows:
  (6) $$\;\;Y=y_{ij}=w_{j}x_{ij}=\left(\begin{array}{ccc} w_{1}x_{11} & \cdots & w_{n}x_{1n}\\ \vdots & \ddots & \vdots \\ w_{1}x_{m1} & \cdots & w_{n}x_{nn} \end{array}\right)$$
  The set of maximum value of each evaluation index is positive ideal scheme${\;A}^{+}$ and the set of minimum value is negative ideal scheme${\;A}^{-}$. The distances from each decision scheme $Q_{i}$ to $A^{+}$and $A^{-}$ are respectively calculated as follows:
  (7) $$D_{i}^{+}={\left[\sum_{j=1}^{n}{\left(y_{ij}-y_{j}^{max}\right)}^{2}\right]}^{1/2}$$
  (8) $$D_{i}^{-}={\left[\sum_{j=1}^{n}{\left(y_{ij}-y_{j}^{min}\right)}^{2}\right]}^{1/2}$$
4. According to the above results, the nearness $Z_{i}$ of each decision scheme can be further calculated:
  (9) $$Z_{i}=D_{i}^{-}/D_{i}^{+}+D_{i}^{-}$$
5. Finally, the final evaluation index of each decision scheme is calculated, which represents the importance of nodes in the network. This paper considers the top five nodes to be the opinion leaders in the community.

#### 4.2.2. Opinion Leader Identification Example

Here, we use an OSP of Local Motors as an example to illustrate the opinion leader identification method. First, a directed, weighted, semantic-based KCN is constructed. Four indexes are calculated using UCINET analysis software and SPSSAU software: node-strength centrality (SC), closeness centrality (CC), betweenness centrality (BC), and structural hole (SH). Then, after the evaluation results of all nodes under the four different indexes are standardized, the original decision matrix is obtained, as shown in Table 4.

The respective information entropies of the four indexes are 0.8855, 0.9465, 0.8745 and 0.9334, the respective weights of these indexes are $w_{SC}=0.318$,$w_{CC}=0.1485$,$w_{BC}=0.3485$ and$\;w_{SH}=0.185$, and the weighted standard decision matrix, *Y*, is:

(10) $$Y=\left[\begin{array}{cc} \begin{array}{cc} 0.0009 & 0.0190\\ 0.0043 & 0.1019\\ 0.0042 & 0.1016 \end{array} & \begin{array}{cc} 0 & 0.00018\\ 0.00038 & 0.00100\\ 0.00021 & 0.00080 \end{array}\\ \begin{array}{cc} 0.0071 & 0.1021\\ 0.0135 & 0.1023\\ 0.0089 & 0.1021 \end{array} & \begin{array}{cc} 0.00320 & 0.00220\\ 0.00160 & 0.00150\\ 0.00170 & 0.00190 \end{array}\\ \begin{array}{cc} 0.0104 & 0.1014\\ 0.0119 & 0.1016\\ \cdots & \cdots \end{array} & \begin{array}{cc} 0.00210 & 0.00190\\ 0.00240 & 0.00420\\ \cdots & \cdots \end{array} \end{array}\right]$$

Next, the positive and negative ideal scheme is generated:

(11) $$A^{+}=\left(0.321,\;0.352\;,\;0.15\;,\;0.187\right)$$

(12) $$A^{-}=\left(0.003,\;0.003,0.001,\;0.002\right)$$

The distance D and closeness Z from each decision scheme to the ideal scheme are calculated. Sorted according to closeness Z, the specific results are shown in Table 5. The top five nodes in the final evaluation are nodes 324, 345, 307, 14, and 321 nodes; these are the opinion leaders of the OSP. Where the first four (324, 345, 307, 14) correspond to the nodes with top five degree centrality and top five betweenness centrality, and the fifth (321) is also included in the top ten nodes of degree and betweenness centrality. This shows that in OPS, designers who have professional knowledge, like to cooperate with others and are in the “intermediary” position have greater influence; most designers are concerned about and follow them.

#### 4.2.3. SIR Propagation Model of Opinion Leaders’ Behavior Influence

Having identified the opinion leaders of the KCN, this section now further describes the influence of the opinion leaders on other nodes, that is, the propagation effect of opinion leaders’ collaborative behavior.

The classical SIR epidemic model [58] considers that the total population is a constant N and there are three different groups: the susceptibles (S), those who have not yet been infected but have a possibility of being infected; the infectives (I), those who have been infected and may infect other individuals; and the removal (R), those who are no longer infected and will not be reinfected. In the KCN of an OSC, the infectives (I) are set as the opinion leader node and the susceptibles (S) are all other individuals who participate in the collaboration. When the opinion leader reduces the intensity of knowledge collaboration, they have a certain chance of being infected. Owing to homogeneity, information default and other reasons, they also reduce the collaboration intensity, that is, behavior degradation. At the same time, there is a certain probability that the infectives (I) will take the initiative to restore the willingness to collaborate and become the removals (R) under the environment of community ecological optimization and altruism.

At time t, the numbers of the three groups are$\;S\left(t\right)$, I$\left(t\right)$, R$\left(t\right)$. The infection probability of susceptibles (S) is λ, and the recovery probability after infection is γ. In KCNs, the infection probability is usually dynamic and tends to decline due to the extension of the infection path and the decrease of time effectiveness. Therefore, the negative exponential model is used to describe the change of infection probability:

(13) $$f\left(t\right)=e^{-\mu \left(t-t_{0}\right)\;}$$

where: μ is the characteristic scaling factor, which is used to describe the reduction characteristics of the infection probability. The standard deviation coefficient is a relative index reflecting the degree of dispersion of vertex strength, so we use its reciprocal to express μ. In the directed weighted network, 〈w〉 is the average weight of the network and $\sigma_{w}$ is the standard deviation of the weight. Therefore, μ is:

(14) $$\mu =\frac{\langle w\rangle }{\sigma_{w}}$$

From the semantic-based KCN model G=( V, E, W), we can get: 〈w〉=7.984 and $\sigma_{w}=22.481$,so μ=0.36,$f\left(t\right)=e^{-0.36\left(t-t_{0}\right)\;}$. Finally, all nodes except for the opinion leader node have recovery behavior. The recovery probability depends on the degree of network information flow, the collaboration needs of nodes, and the community’s investment in node recovery resources. Therefore, to facilitate the calculation, the overall recovery probability is set as γ=0.1. In summary, the SIR model equations of opinion leaders are:

(15) $$\left\{\begin{array}{c} \frac{dI\left(t\right)}{dt}=e^{-0.36\left(t-t_{0}\right)\;}I\left(t\right)S\left(t\right)-0.1I\left(t\right)\\ \frac{dS\left(t\right)}{dt}=-e^{-0.36\left(t-t_{0}\right)\;}I\left(t\right)S\left(t\right)\;\;\;\;\;\;\;\;\;\;\;\;\;\;\;\;\\ \frac{dR\left(t\right)}{dt}=0.1I\left(t\right)\;\;\;\;\;\;\;\;\;\;\;\;\;\;\;\;\;\;\; \end{array}\right.$$

## 5. Dynamic Robustness Analysis of Knowledge Collaborative Network

### 5.1. Robustness Evaluation Index

Network robustness can be defined as the degree of retention of network performance when network nodes or edges fail [59]. The impact of such node or edge failure for the KCN of an OSC includes (1) the destruction of network connectivity, which reduces knowledge collaborative intensity, and (2) a decrease in network efficiency, which increases the difficulty of knowledge collaboration. As such, the robustness evaluation index proposed in this paper includes both network connectivity and weighted efficiency.

#### 5.1.1. Relative Size of Network Connectivity S

To reflect the degree of network connectivity retention after the network is attacked, the relative network connectivity size S is defined as the relative size of the largest connected subgraph node intensity of the network:

(16) $$S=\frac{S_{lc}^{\prime }}{S_{lc}}$$

where: $S_{lc}^{\prime }$ is the sum of the node intensity of the maximum connected subgraph of the network after being attacked and $S_{lc}$ is the sum of the node intensity of the original network. The calculation formula for the sum of node intensity is

(17) $$S_{lc}=\overset{N}{\underset{i}{\sum }}\overset{N}{\underset{j}{\sum }}w_{ij}$$

where: N is the total number of nodes in the network and $w_{ij}$ is the edge weight of nodes i and j. In the weighted KCN of the OSC, node intensity represents the knowledge collaborative intensity. Therefore, the smaller the value of S, the greater the decrease in knowledge collaborative intensity after the network is attacked (i.e., the lower the robustness of connectivity), and vice versa. 

#### 5.1.2. Relative Size of Weighted Efficiency H

Network efficiency describes the difficulty of information dissemination. Network efficiency is expressed as the sum of the efficiency of all nodes in the network, where node efficiency is the reciprocal of the shortest path length between two nodes [60]:

(18) $$E=\frac{1}{n\left(n-1\right)}\underset{i\neq j}{\sum }\frac{1}{d_{ij}}$$

where $d_{ij}$ is the distance between nodes i and j, and n is the number of nodes. Weighted efficiency is based on the weighted shortest paths notion. For weighted networks, a path between two nodes is the sum of the weight associated to the links necessary to travel between the nodes [61]; consequently, the directed weighted shortest path, expressed as$\;{\left(d_{w}\right)}_{ij}$, is the minimum sum of the weights necessary to travel form nodes i to j. When the $w_{ij}$ of edge $e_{ij}$ is defined as dissimilarity weight, such as “costs’’, the WSP is the minimum sum of the weight of the links to travel between the nodes, that is,$\;{\left(d_{w}\right)}_{i,j}=w_{ij}$; when the $w_{ij}$ of edge $e_{ij}$ is defined as similarity weight, such as “flows’’, the WSP is the minimum value of the reciprocal sum of edge weight on the links, that is, dwi,j=1wij .

From the above analysis, the weighted efficiency$\;E_{w}$ of the directed weighted network can be obtained:

(19) $$E_{G}=\frac{1}{n\left(n-1\right)}\underset{i\neq j}{\sum }\frac{1}{{\left(d_{w}\right)}_{ij}}$$

It can be seen from part 3.1 that the weight of KCN is similarity weight, which is "flows", therefore, wheredwij=1wij .

To reflect the degree of knowledge collaborative efficiency retention after the network is attacked, the relative knowledge collaborative efficiency size H is defined as follows:

(20) $$H=\frac{E_{w}^{\prime }}{E_{w}}$$

where $E_{G}^{\prime }$ is the weighted efficiency of the attacked network and $E_{G}\;$is the weighted efficiency of the original network. The value range of H is$\left[0,1\right]$. When H=0, the network efficiency drops to its lowest after the attack, that is, designers in the network do not have any form of collaboration. When H=1, the efficiency of the whole network remains at the original level, without any impact on the network efficiency due to the failure of edge weights.

### 5.2. Degradation Mode Design of Knowledge Collaboration Behavior

Degradation mode design is the key to robustness analysis. For the open-source design community, the KCN will not be subject to external attacks. The main risks and disadvantages of the network come from (1) designers voluntarily leaving the community, which is called node degradation, and (2) designers’ willingness to collaborate is reduced, which is called the degradation of knowledge collaboration behavior. Most existing research is based on the degradation of the nodes themselves, and little research exists on the degradation of knowledge collaboration behavior or the propagation mode of opinion leaders’ behavior degradation in the community. In OSCs, high-strength nodes are very important for maintaining network connectivity, and they represent the main knowledge contribution nodes. The high betweenness node plays an important role in maintaining the information dissemination speed of the network, and it represents the main knowledge dissemination node in the network. By ranking nodes according to their point strength and betweenness, and reducing the weights of the directed edges in turn, we can describe the collective degradation of the main knowledge contribution behavior and the main knowledge dissemination behavior.

**Figure 2:**
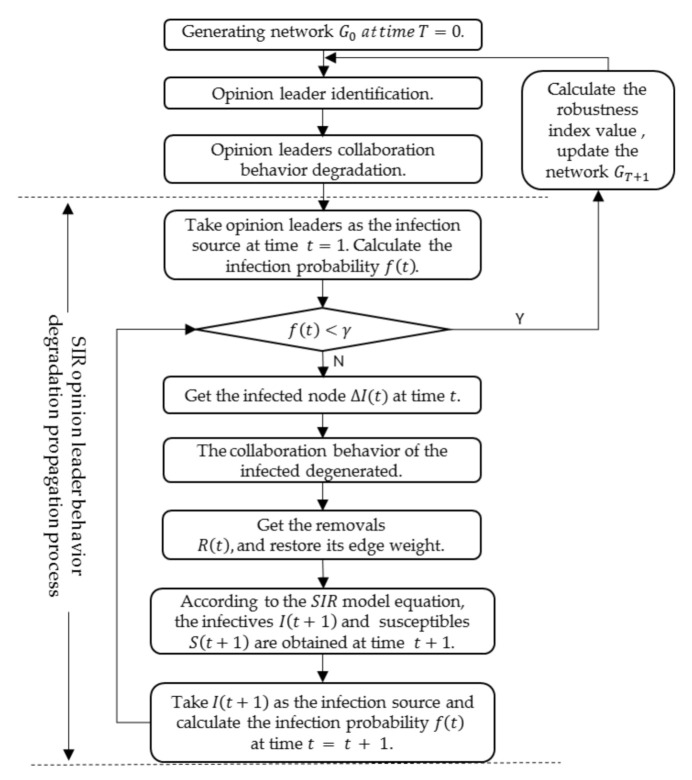
The simulation process of opinion leaders’ collaborative behavior degradation.

In addition, opinion leaders are the most influential nodes in the network. Under the influence of the external environment, dissatisfaction with the community ecology and their own factors, their knowledge collaborative intensity may decrease; if so, their propagation characteristics lead to the degradation of other nodes’ collaborative behavior. Therefore, this should be reflected in the design of the degradation mode of opinion leaders’ collaborative behavior. In this paper, an SIR model is used to simulate the propagation mode of the opinion leaders’ degradation behavior and calculate the change of network performance. The simulation process is shown in Figure 2. 

Considering the characteristics of the above three kinds of nodes and their roles in the network, we design three degradation modes for collaborative behavior, as shown in Table 6. Here, ε represents the degradation degree of the collaborative behavior, known as the “degradation coefficient”.

### 5.3. Simulation Analysis on Dynamic Robustness of KCN

Based on the construction of a semantic-based KCN, the identification of opinion leaders, and the design of a knowledge collaboration behavior degradation mode, the index changes of network robustness under random degradation, and the three degradation patterns are simulated using Python 3.7 software. Originpro 9.0 software is used to create the contrast chart of the experimental results.

**Figure 3:**
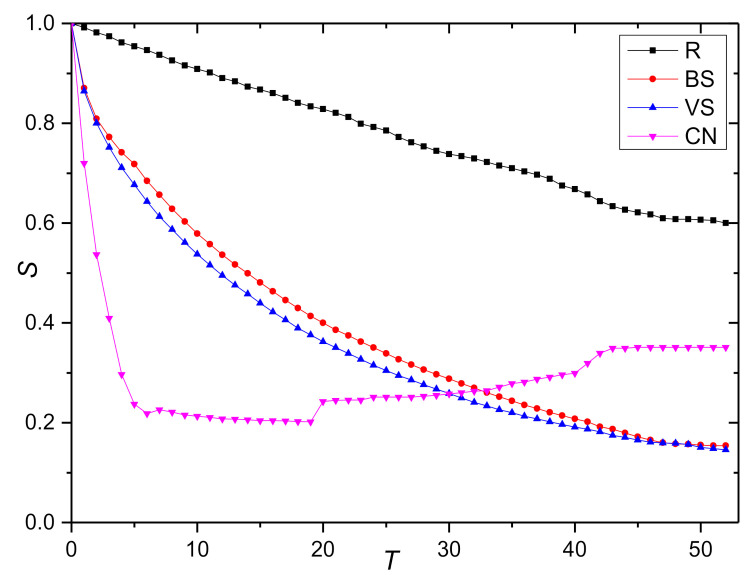
The change trend of the relative size of network connectivity S.

**Figure 4:**
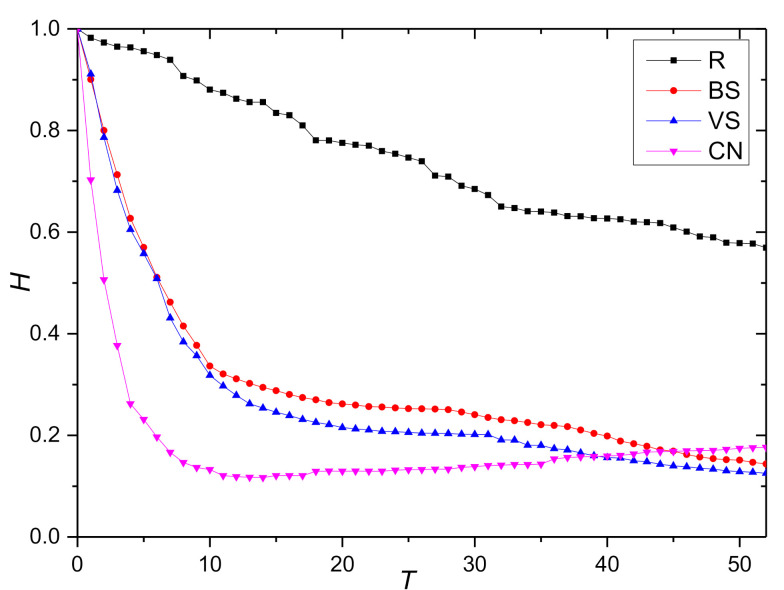
The change trend of the relative size of weighted efficiency H.

This paper starts from T=0 to simulate the change of network robustness index during the continuous decline of collaborative behavior. To observe the influence of knowledge collaboration behavior degradation on network robustness more clearly, the degradation coefficient is taken as ε=0.8. In the propagation process of the degradation of opinion leaders’ collaborative behavior, the initial time is set at$\;t_{0}=0$. Figure 3 shows the change trend of the relative size of network connectivity under the behavior degradation mode, and Figure 4 shows the change trend of the relative size of weighted efficiency. Note that the index value rises slightly with the continuous evolution; this is because of the continuous recovery of nodes under the propagation mode of the degradation of opinion leaders’ collaborative behavior. In addition, the curve trend of the other three behavior degradation modes is basically the same. The marginal nodes with a strength of 1 account for the largest proportion in the network. As such, these nodes are more likely to be selected in the random degradation mode. These kinds of nodes have a weak collaboration tendency and a single information exchange channel; as such, the degree of change of the two indexes is the lowest. In contrast, the degree of change of the index value is stronger under the three modes of main knowledge dissemination behavior (BS), main knowledge contribution behavior (VS), and opinion leaders’ collaborative behavior (CN).

The paired T-test is conducted on the change trend data of the index values under the four behavioral degradation modes collected from the simulation experiment, as shown in Table 7. These test results show that the index values under different degradation modes are significantly different. Based on the above analysis, it can be concluded that the random degradation mode has the highest robustness, followed by the degradation mode of main knowledge dissemination behavior, main knowledge contribution behavior, and opinion leaders’ collaborative behavior, namely R > BS > VS > CN.

Through further observation of the change of the index value we find that when t=5, the relative size of network connectivity and weighted efficiency under the degradation mode of opinion leaders’ collaborative behavior (CN) decreased by 80%, while the index value under the degradation modes of main knowledge dissemination behavior (BS) and main knowledge contribution decreased by only 40%. On the one hand, this result confirms that the opinion leaders identified by the method in Section 3 do have a strong influence. On the other hand, it shows that protecting the opinion leader nodes to avoid their behavior degradation is the key to keeping network performance and improving the robustness of the KCN.

Due to the following effect of opinion leaders on other designers, the index value of the network under the degradation mode of opinion leaders’ collaborative behavior drops to 50% of the original network at time T=2 and reaches its lowest value at time T=5. Then, for a long time, the network performance remains at its lowest state. With the evolution of the network, knowledge collaboration behavior constantly recovers, which makes the network performance continuously improve. The relative size of network connectivity can recover 17% on the basis of the lowest value, while the relative size of weighted efficiency is only about 5%, which indicates that the majority of immune nodes are marginal nodes and the heterogeneity is not obvious.

When comparing the degradation patterns of main knowledge contribution behavior (VS) and main knowledge dissemination behavior (BS), we find that the top five nodes in the original network partially overlap and the decline degree of the initial index value is basically the same. With the continuous evolution of the network, the index value is more sensitive to the degradation of the main knowledge contribution behavior; therefore, protecting the main knowledge contribution nodes should also be the focus of robust management.

## 6. Conclusions

Based on the characteristics of knowledge collaboration in OSCs, we analyzed the network robustness under the degeneration model of knowledge collaboration behavior. Our research contributions are as follows:

1. A semantic-based KCN was established by taking the weighted processing of collaboration content and collaboration frequency among designers in the OSD community as edge weights.
2. The index system of strength centrality, closeness centrality, betweenness centrality, and structure hole was constructed. The multi-attribute comprehensive evaluation method of entropy weight and TOPSIS was used to identify the opinion leaders, and the SIR model of opinion leader’s behavior influence was proposed to describe the propagation of opinion leader’s behavior.
3. Based on the behavior characteristics of designers in the OSD community, we designed three degradation modes based on opinion leaders’ collaborative behavior, main knowledge dissemination behavior and main knowledge contribution behavior. Robustness analysis experiments were conducted based on empirical data from the Local Motors OSD community. The analysis results showed that network robustness was the lowest under the degradation mode of opinion leaders’ collaborative behavior, followed by the degradation modes of main knowledge contribution behavior and main knowledge dissemination behavior. The degradation mode of random behavior had the highest robustness. Under the degradation mode of opinion leaders’ collaborative behavior and the propagation mode of their degraded behaviors, network performance can quickly drop to its lowest point. The continuous restoration of collaborative behaviors by nodes can cause the network performance to rise after a sustained period at its lowest state, but this increase is limited.

Based on the analysis results, the following management implications were also obtained:

1. The community should effectively identify the nodes of opinion leaders and encourage the knowledge collaboration behavior of such nodes with core influence. Research shows that a harmonious community environment, perfect incentive mechanism, and competitive coordination mechanism can effectively improve the collaboration behavior of nodes [62,63]. The community can establish designer privacy protection, clear intellectual property rights, and other systems. Opinion leaders could be given more incentive measures, such as resource authority, identity authentication, and privilege level. Further, increased opportunities could be given for online meetings, offline development, and other activities. At the same time, community managers should ensure timely information guidance to avoid behavior propagation caused by information deficit. For example, the setting of mechanisms such as timely information release, status updates and intelligent Q and As.
2. Community managers should also pay attention to the protection of main knowledge contribution designers and main knowledge dissemination designers to avoid the degradation of their collaborative behavior. For example, the star sign should be set for the high-quality knowledge contributed by designers, and the case databases should be set up for the active users to consult and so on; this should increase the sense of worth of the main knowledge contributors. Further, the main knowledge dissemination designers should be encouraged to join in with the community management work, and attention should be paid to their opinions on the development of the community to increase their sense of belonging and achievement.
3. A talent introduction mechanism should be set to attract more designers to join the community to collaborate knowledge. Further, after the degradation of designers’ behaviors, resources should be first concentrated to restore the collaborative behaviors of core influential nodes to reduce the rapid decline of network performance.

This paper constructed a semantic-based KCN to effectively identify opinion leaders and studied the network robustness under different behavior degradation modes. This method is particularly suited to organizations that prioritize large-scale knowledge collaboration, such as OSP or OSD communities. One limitation is that our work is aimed at the single project network. In the OSC, the same designer can participate in multiple projects, and the degradation of designers’ collaborative behavior may have an impact on multiple projects. Therefore, we will carry out further research on the robustness of the OSC’s multi-project KCN in the future.

## Figures and Tables

**Table 1:** Network topology parameters and network characteristics of the semantic-based KCN.

Topological Parameter						Network Characteristic		
Number of nodes	Average out-degree	Average path length	Clustering coefficient	Network efficiency	Small world parameter	Small world characteristic	Scale free property	Assortativity
463	7.984	2.6403	0.535	0.2718	23.2537	Yes	Yes	No

Note: According to Davis, Yoo and Baker [50], the small-world parameters can be expressed as:$\;SW=\left[Cactual/Lactual]\;*[Lrandom/Crandom\right]$, where: Cactual is the average clustering coefficient of the network, Lactual is the average path length, $Lrandom=ln\left(n\right)/ln\left(‹k›\right),Crandom=‹k›/n,\;n$ is the number of nodes and ‹k› is the average degree. The network efficiencyis: $E=\frac{1}{n\left(n-1\right)}\underset{i\neq j}{\sum }\frac{1}{d_{ij}}\;,\mathrm{where}:d_{ij}\;$is length of the weighted shortest path from node i to j [51].

**Table 2:** Top ten node list for strength centrality, closeness centrality, and betweenness centrality.

	Strength Centrality				Closeness Centrality				Betweenness Centrality	
	Node	Out	Node	In	Node	In	Node	Out	Node	Betweenness
1	324	269.69	324	157.44	206	0.592	38	0.258	324	55712.934
2	345	204.84	345	113.09	436	0.535	87	0.241	345	24626.021
3	307	155.43	307	95.768	369	0.478	233	0.238	307	13746.778
4	208	144.83	14	79.208	45	0.476	262	0.236	14	8258.913
5	14	142.88	350	71.493	403	0.474	393	0.230	350	7317.06
6	321	110.1	321	68.513	72	0.439	143	0.220	47	7098.686
7	313	74.93	206	68.367	152	0.411	180	0.217	208	4458.465
8	90	70.83	313	63.012	441	0.401	361	0.216	321	4360.754
9	109	67.46	269	58.6	134	0.394	444	0.187	82	3249.533
10	127	62.14	420	51.842	363	0.393	266	0.168	313	3088.124

**Table 3:** Top ten node list of structural hole hierarchy.

Node	Hierarchy	Effective Size	Efficiency	Constraint
112	0.001	2	1	0.5
120	0.001	2	1	0.5
320	0.001	2	1	0.5
84	0.001	1.506	0.502	0.928
357	0.001	1.156	0.578	1.062
57	0.001	1	0.5	1.364
260	0.001	1	0.5	1.367
191	0.001	1	0.5	1.412
158	0.001	1	0.5	1.422
140	0.002	1	0.5	1.371

**Table 4:** Four index standardization matrix.

	Index	SC	CC	BC	SH
Node					
1		0.0029	0.12819	0	0.001
2		0.0138	0.68665	0.001	0000529
3		0.0134	0.68427	0.006	0.00406
4		0.0222	0.68783	0.0092	0.01190
5		0.0425	0.68902	0.0049	0.00806

**Table 5:** Opinion leader identification results from the Local Motors example.

Node	$D_{i}^{+}$	$D_{i}^{-}$	$Z_{i}$
324	0.132	0.486	0.786
345	0.232	0.316	0.576
307	0.307	0.254	0.453
14	0.342	0.237	0.409
321	0.376	0.225	0.374

**Table 6:** Degradation mode design of knowledge collaboration behavior.

	Degradation Mode Description	Degradation Simulation Calculation Process
Degradation mode of knowledge Collaboration behavior	The degradation of opinion leaders’ collaborative behavior (CN)	(1) The directed edge weight of the five identified opinion leaders in the network is reduced to the original weight ε times. (2) Taking the opinion leader node as the initial infection source into the SIR model, the weight of the infected node is reduced to the original value ε times. At the end of evolution, the robustness index value is calculated. Take the network at this time as the current network, then identify the opinion leader node and repeat the above two steps. Repeat n times to simulate the most influential node and its degradation behavior propagation mode. The simulation process is shown in Figure 2.
	The degradation of main knowledge contribution behavior (VS)	Sort the nodes generated by the network according to out-strength. Based on the sorting result, select the top five nodes to reduce the weight of the directed edge to the original value ε times. Take the network at this time as the current network, then calculate the out-strength of nodes and sort to reduce the directed edge weights of the first five nodes. Repeat n times to simulate the degradation of main knowledge collaboration behavior.
	The degradation of main knowledge dissemination behavior (BS)	Sort the nodes generated by the network according to node betweenness. Based on the sorting result, select the top five nodes to reduce the weight of the directed edge to the original value ε times. Take the network at this time as the current network, then calculate the node betweenness of nodes and sort to reduce the directed edge weights of the first five nodes. Repeat n times to simulate the degradation of main knowledge dissemination behavior.
Random degradation	Random degradation of collaborative behavior (R)	Randomly select five nodes to reduce the weight of the directed edge to the original value ε times. Repeat n times to simulate the random degradation of collaborative behavior.

**Table 7:** Paired sample T-test under different behavioral degradation modes.

	Paired Degradation Mode	M	SD	95% Confidence		t	df	Sig
				Lower Limits	Upper Limits			
	R-BS	0.448954	0.012223	0.424437	0.473481	36.745	52	0.000
*H*	BS-VS	0.033671	0.001818	0.030032	0.037324	18.544	52	0.000
	VS-CN	0.087575	0.014925	0.057614	0.117548	5.868	52	0.000
	R-BS	0.392329	0.014702	0.362827	0.421832	26.685	52	0.000
*S*	BS-VS	0.025411	0.002538	0.020317	0.030041	10.012	52	0.000
	VS-CN	0.063971	0.027419	0.008948	0.118992	2.333	52	0.024

## Data Availability

The used and analyzed datasets during the present study are available from the corresponding author on reasonable request.

## Abbreviations

The following abbreviations are used in this manuscript:

KCN	Knowledge Collaborative Network
OSP	Open source project
OSD	Open source design
WSP	weighted shortest path
CN	The degradation of opinion leaders’ collaborative behavior
VS	The degradation of main knowledge contribution behavior
BS	The degradation of main knowledge dissemination behavior
R	Random degradation of collaborative behavior

## References

[1] Hu Y., Zhou H. (2019). Open source design community simulation model research based on behavior data. Comput. Integr. Manuf. Syst..

[2] Pan Y., Xu Y.C., Wang X., Zhang C., Ling H., Lin J. (2015). Integrating social networking support for dyadic knowledge exchange: A study in a virtual community of practice. Inf. Manag..

[3] O’Mahony S. (2003). Guarding the commons: How community managed software projects protect their work. Res. Policy.

[4] Xu B.C., Zhang X.D. (2019). Opinion leader identification and following effect simulation in the open source community. Inf. Stud. Theory Appl..

[5] Zhou H.L. (2018). Research on Dynamic Robustness of Knowledge Collaboration Network of Open Source Product Community. Ph.D. Thesis.

[6] Mohammadi S.A., Andalib A. Using the opinion leaders in social networks to improve the cold start challenge in recommender systems. Proceedings of the 3rd International Conference on Web Research (ICWR).

[7] Lees-Marshment J., Helms L. (2012). Political marketing and opinion leadership: Comparative perspectives and findings. Comparative Political Leadership.

[8] Gentina E., Kilic D., Dancoine P.-F. (2017). Distinctive role of opinion leaders in the social networks of school adolescents: An investigation of e-cigarette use. Public Health.

[9] Koeslag-Kreunen M.G., van der Klink M.R., Bossche P.V.D., Gijselaers W. (2018). Leadership for team learning: The case of university teacher teams. High. Educ..

[10] Oueslati W., Arrami S., Dhouioui Z., Massaabi M. (2021). Opinion leaders’ detection in dynamic social networks. Concurr. Comput. Pr. Exp..

[11] Anagnostopoulos A., Kumar R., Mahdian M. Influence and correlation in social networks. Proceedings of the 14th ACM SIGKDD International Conference on Knowledge Discovery and Data Mining—KDD 08.

[12] Zhang B., Bai Y., Zhang Q., Lian J., Li M. (2020). An opinion-leader mining method in social networks with a phased-clustering perspective. IEEE Access.

[13] Mohammadaghdam S., Navimipour N.J. (2016). Opinion leaders selection in the social networks based on trust relationships propagation. Karbala Int. J. Mod. Sci..

[14] Moqri M., Mei X., Qiu L., Bandyopadhyay S. (2018). Effect of “following” on contributions to open source communities. J. Manag. Inform. Syst..

[15] Deshpande A., Riehle D., Russo B., Damiani E., Hissam S., Lundell B., Succi G. (2008). The total growth of open source. Open Source Development, Communities and Quality, Proceedings of the IFIP 20th World Computer Congress, Working Group 2.3 on Open Source Software, Milano, Italy, 7–10 September 2008.

[16] Rashid M., Clarke P.M., O’Connor R.V. (2019). A systematic examination of knowledge loss in open source software projects. Int. J. Inf. Manag..

[17] Shi L., Cheng Y., Shao J., Wang X., Xu Y. (2020). A novel nonlinear leader-follower opinion dynamics model with asynchronous trust/distrust evolution. arXiv.

[18] Vodopivec N., Adam C., Chanteau J.P. (2021). Modeling opinion leader’s role in the diffusion of innovation. arXiv.

[19] Quinn K.G. (2020). Applying the popular opinion leader intervention for HIV to COVID-19. AIDS Behav..

[20] Bonacich P. (1972). Factoring and weighting approaches to status scores and clique identification. J. Math. Sociol..

[21] Freeman L.C. (1978). Centrality in social networks conceptual clarification. Soc. Netw..

[22] Ren Z., Liu J., Feng S., Hu Z., Qiang G. (2013). Analysis of the spreading influence of the nodes with minimum K-shell value in complex networks. Acta Phys. Sin. Chin. Ed..

[23] Zhu Z., Cui Z., Ding X., Rui M. (2017). Identifying opinion leaders in major sudden public opinion spread based on entropy-weighted grey correlation model. J. China Soc. Sci. Tech. Inf..

[24] Jain L., Katarya R., Sachdeva S. Role of Opinion Leader for the diffusion of products using Epidemic model in Online Social Network. Proceedings of the 2019 Twelfth International Conference on Contemporary Computing (IC3).

[25] Chen Y.-C. (2018). A novel algorithm for mining opinion leaders in social networks. World Wide Web.

[26] Chen Y., Wang X., Tang B., Xu R., Yuan B., Xiang X., Bu J. Identifying opinion leaders from online comments. Proceedings of the Social Media Processing: Third National Conference, SMP 2014.

[27] Jiang L., Ge B., Xiao W., Gao M. BBS opinion leader mining based on an improved PageRank algorithm using MapReduce. Proceedings of the 2013 Chinese Automation Congress (CAC 2013).

[28] Li C., Bai J., Zhang L., Tang H., Luo Y. (2019). Opinion community detection and opinion leader detection based on text information and network topology in cloud environment. Inf. Sci..

[29] Li C., Bai J., Wenjun Z., Xihao Y. (2019). Community detection using hierarchical clustering based on edge-weighted similarity in cloud environment. Inf. Process. Manag..

[30] Wang C., Du Y.J., Tang M.W. Opinion leader mining algorithm in microblog platform based on topic similarity. Proceedings of the 2nd IEEE International Conference on Computer and Communications (ICCC).

[31] Ye H., Du J. Opinion leader mining of social network combined with hierarchical sentiment analysis. Proceedings of the Chinese Intelligent Automation Conference.

[32] Atienza-Barthelemy J., Martin-Gutierrez S., Losada J.C., Benito R.M. (2019). Relationship between ideology and language in the Catalan independence context. Sci. Rep..

[33] Shao P., Chen H. (2019). Driving factors for opinion diffusion behavior in consumers on online social networks: A study of network characteristics. IEEE Access.

[34] Jain L., Katarya R. (2019). Discover opinion leader in online social network using firefly algorithm. Expert Syst. Appl..

[35] Katarya R., Gautam D. Survey on opinion leader in social network using data mining. Proceedings of the 5th International Conference on Advanced Computing & Communication Systems (ICACCS).

[36] Wang X.N., Zhu Z.G. (2017). Research on the law of view information communication based on improved SIR model in social network. Inf. Sci..

[37] Zhang W.D., Li S.T., Liang E.P. (2019). Research on social media user following behavior based on complete information game model. Inf. Sci..

[38] Xiong X., Ma J., Wang M., Zhou G., Xu K. (2015). Information diffusion model in modular microblogging networks. World Wide Web.

[39] Zhou H.-L., Zhang X.-D. (2018). Dynamic robustness of knowledge collaboration network of open source product development community. Phys. A Stat. Mech. Appl..

[40] Albert R., Jeong H., Barabasi A. (2000). Error and attack tolerance of complex networks. Nature.

[41] Hao Y., Jia L., Wang Y. (2019). Robustness of weighted networks with the harmonic closeness against cascading failures. Phys. A Stat. Mech. Appl..

[42] Wang S., Liu J. (2016). Robustness of single and interdependent scale-free interaction networks with various parameters. Phys. A Stat. Mech. Appl..

[43] Fuge M., Tee K., Agogino A., Maton N. (2014). Analysis of collaborative design networks: A case study of openideo. J. Comput. Inf. Sci. Eng..

[44] Donadelli S.M. (2015). The Impact of Knowledge Loss on Software Projects: Turnover, Customer Found Defects, and Dormant Files. M.A. Dissertation.

[45] Zhou H., Zhang X., Hu Y. (2020). Robustness of open source product innovation community’s knowledge collaboration network under the dynamic environment. Phys. A Stat. Mech. Appl..

[46] Gamalielsson J., Lundell B. (2014). Sustainability of open source software communities beyond a fork: How and why has the LibreOffice project evolved?. J. Syst. Softw..

[47] Schweitzer F., Mavrodiev P., Seufert A.M., Garcia D. (2020). Modeling User reputation in online social networks: The role of costs, benefits, and reciprocity. Entropy.

[48] Lei S., Zhang X., Xie S., Zheng X. (2021). Dynamic robustness of semantic-based collaborative knowledge network of open source project. Entropy.

[49] Rose S.J., Engel D., Cramer N., Cowley W.E. (2010). Automatic Keyword Extraction from Individual Documents.

[50] Davis G.F., Yoo M., Baker W.E. (2003). The small world of the American corporate elite, 1982–2001. Strat. Organ..

[51] Bellingeri M., Bevacqua D., Scotognella F., Cassi D. (2019). The heterogeneity in link weights may decrease the robustness of real-world complex weighted networks. Sci. Rep..

[52] Iyer S., Killingback T., Sundaram B., Wang Z. (2013). Attack robustness and centrality of complex networks. PLoS ONE.

[53] Huang J. Combining entropy weight and TOPSIS method for information system selection. Proceedings of the IEEE Conference on Cybernetics and Intelligent Systems.

[54] Yu H., Liu Z., Li Y. (2013). Key nodes in complex networks identified by multi-attribute decision-making method. Acta Phys. Sin..

[55] Beauchamp M.A. (1965). An improved index of centrality. Syst. Res. Behav. Sci..

[56] Freeman L.C. (1977). A set of measures of centrality based on betweenness. Sociometry.

[57] Burt R.S. (2004). Structural holes and good ideas. Am. J. Sociol..

[58] Pastor-Satorras R., Vespignani A. (2001). Epidemic spreading in scale-free networks. Phys. Rev. Lett..

[59] Crucitti P., Latora V., Marchiori M., Rapisarda A. (2003). Efficiency of scale-free networks: Error and attack tolerance. Phys. A Stat. Mech. Appl..

[60] Wang J., Wu X., Chen Y. (2013). Invulnerability simulation of weighted complex networks with different information. J. Cent. South Univ. Sci. Technol..

[61] Bellingeri M., Cassi D. (2018). Robustness of weighted networks. Phys. A Stat. Mech. Appl..

[62] Killingsworth B., Xue Y., Liu Y. (2016). Factors influencing knowledge sharing among global virtual teams. Team Perform. Manag. Int. J..

[63] Corvello V., Chimenti M., Giglio C., Verteramo S. (2020). An investigation on the use by academic researchers of knowledge from scientific social networking sites. Sustainability.

